# 2-Cyano-*N*′-[1-(2-hy­droxy­phen­yl)ethyl­idene]acetohydrazide monohydrate

**DOI:** 10.1107/S1600536811042565

**Published:** 2011-10-22

**Authors:** Hongbo Li, Peng Chen, Zhonglu You

**Affiliations:** a College of Chemistry and Biology Engineering, Yancheng Institute of Technology, Yancheng 224051, People’s Republic of China; b Department of Chemistry and Chemical Engineering, Liaoning Normal University, Dalian 116029, People’s Republic of China

## Abstract

The title compound, C_11_H_11_N_3_O_2_·H_2_O, was obtained by the reaction of 2-acetyl­phenol with cyano­acetohydrazide in methanol. The asymmetric unit contains two hydrazone mol­ecules and two water mol­ecules of crystallization. There is an intra­molecular O—H⋯N hydrogen bond in each hydrazone mol­ecule. The crystal structure is stabilized by inter­molecular N—H⋯O, O—H⋯O and O—H⋯N hydrogen bonds.

## Related literature

For the structures of hydrazones, see: Wang *et al.* (2011 ▶); Hashemian *et al.* (2011 ▶); Singh & Singh (2010 ▶); Ahmad *et al.* (2010 ▶). For compounds we have reported on recently, see: Li & Ni (2011 ▶); Li & Chen (2011 ▶).
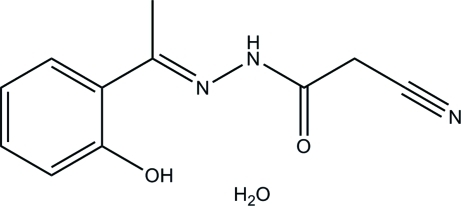


## Experimental

### 

#### Crystal data

C_11_H_11_N_3_O_2_·H_2_O
*M* *_r_* = 235.24
Monoclinic, 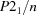

*a* = 17.387 (3) Å
*b* = 7.576 (2) Å
*c* = 17.855 (3) Å
β = 90.962 (2)°
*V* = 2351.7 (8) Å^3^
*Z* = 8
Mo *K*α radiation
μ = 0.10 mm^−1^
*T* = 298 K
0.20 × 0.18 × 0.17 mm

#### Data collection

Bruker SMART 1K CCD area-detector diffractometer
Absorption correction: multi-scan (*SADABS*; Sheldrick, 2004 ▶) *T*_min_ = 0.981, *T*_max_ = 0.983
14877 measured reflections
4994 independent reflections
2551 reflections with *I* > 2σ(*I*)
*R*_int_ = 0.060

#### Refinement

*R*[*F* ^2^ > 2σ(*F* ^2^)] = 0.070
*wR*(*F* ^2^) = 0.147
*S* = 1.02
4994 reflections
329 parameters
8 restraints
H atoms treated by a mixture of independent and constrained refinement
Δρ_max_ = 0.19 e Å^−3^
Δρ_min_ = −0.18 e Å^−3^

### 

Data collection: *SMART* (Bruker, 2001 ▶); cell refinement: *SAINT* (Bruker, 2001 ▶); data reduction: *SAINT*; program(s) used to solve structure: *SHELXTL* (Sheldrick, 2008 ▶); program(s) used to refine structure: *SHELXTL*; molecular graphics: *SHELXTL*; software used to prepare material for publication: *SHELXTL*.

## Supplementary Material

Crystal structure: contains datablock(s) I, global. DOI: 10.1107/S1600536811042565/qm2036sup1.cif

Structure factors: contains datablock(s) I. DOI: 10.1107/S1600536811042565/qm2036Isup2.hkl

Supplementary material file. DOI: 10.1107/S1600536811042565/qm2036Isup3.cml

Additional supplementary materials:  crystallographic information; 3D view; checkCIF report

## Figures and Tables

**Table 1:** Hydrogen-bond geometry (Å, °)

*D*—H⋯*A*	*D*—H	H⋯*A*	*D*⋯*A*	*D*—H⋯*A*
O6—H6*B*⋯N6^i^	0.84 (1)	2.17 (2)	2.975 (4)	159 (3)
O5—H5*B*⋯O3^ii^	0.85 (1)	2.43 (2)	3.120 (4)	140 (3)
O6—H6*A*⋯O4^iii^	0.85 (1)	2.06 (1)	2.900 (3)	173 (3)
O5—H5*A*⋯O2	0.85 (1)	1.93 (1)	2.777 (3)	174 (3)
N5—H5⋯O5^iv^	0.90 (1)	1.93 (1)	2.820 (3)	171 (3)
N2—H2⋯O6^v^	0.90 (1)	2.03 (1)	2.905 (3)	162 (3)
O3—H3*A*⋯N4	0.82	1.82	2.534 (3)	145
O1—H1⋯N1	0.82	1.81	2.528 (3)	145

Symmetry codes: (i) 
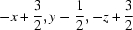
; (ii) 
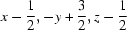
; (iii) 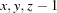; (iv) 
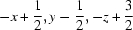
; (v) 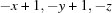.

**Fig. 1.**
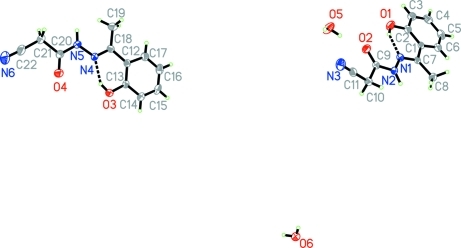
The molecular structure of (I), with atom labels and 30% probability displacement ellipsoids for non-H atoms. Intramolecular O—H···N hydrogen bonds are shown as dashed lines.

**Fig. 2.**
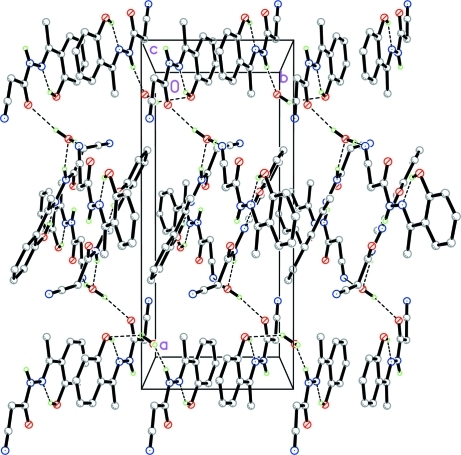
The packing of (I), viewed down the c axis. Hydrogen bonds are shown as dashed lines. H atoms not involved in hydrogen bonding have been omitted.

## supplementary crystallographic information

### Comment

Recently, a great number of hydrazones derived from the reaction of salicylaldehyde and its derivatives with benzohydrazides have been reported (Wang *et al.*, 2011; Hashemian *et al.*, 2011; Singh & Singh, 2010; Ahmad *et al.*, 2010). As a continuation of our work on the hydrazones derived from cyanoacetohydrazide (Li & Ni, 2011; Li & Chen, 2011), in this paper, the title new hydrazone compound, (I), is reported.

The asymmetric unit of the compound contains two hydrazone molecules and two water molecules of crystallization, Fig. 1. There is an intramolecular O—H···N hydrogen bond (Table 1) in the hydrazone molecule. The non-hydrogen atoms of the hydrazone molecules are approximately coplanar, with mean deviations from the least-squares planes of 0.107 (3) Å for molecule A and 0.100 (3) Å for molecule B. The crystal structure is stabilized by intermolecular N—H···O, O—H···O, and O—H···N hydrogen bonds (Table 2, and Fig. 2).

### Experimental

The title compound was obtained by the reaction of equimolar quantities (1.0 mmol each) of 2-acetylphenol with cyanoacetohydrazide in methanol. Single crystals suitable for X-ray diffraction were obtained by the slow evaporation of the solution containing the compound in open air.

### Refinement

The water H atoms and the amino H atoms were located from a difference Fourier map and refined isotropically, with O—H, H···H, and N—H distances restrained to 0.85 (1), 1.37 (2), and 0.90 (1) Å, respectively. All other H atoms were positioned geometrically and refined using a riding model, with C—H = 0.93–0.97 Å, and with *U*_iso_(H) = 1.2 (1.5 for methyl group) times *U*_eq_(C).

### Crystal data

**Table:**

C_11_H_11_N_3_O_2_·H_2_O	*F*(000) = 992
*M**_r_* = 235.24	*D*_x_ = 1.329 Mg m^−^^3^
Monoclinic, *P*2_1_/*n*	Mo *K*α radiation, λ = 0.71073 Å
*a* = 17.387 (3) Å	Cell parameters from 2383 reflections
*b* = 7.576 (2) Å	θ = 2.3–24.5°
*c* = 17.855 (3) Å	µ = 0.10 mm^−^^1^
β = 90.962 (2)°	*T* = 298 K
*V* = 2351.7 (8) Å^3^	Block, colorless
*Z* = 8	0.20 × 0.18 × 0.17 mm

### Data collection

**Table:**

Bruker SMART 1K CCD area-detector diffractometer	4994 independent reflections
Radiation source: fine-focus sealed tube	2551 reflections with *I* > 2σ(*I*)
graphite	*R*_int_ = 0.060
ω scans	θ_max_ = 27.0°, θ_min_ = 2.3°
Absorption correction: multi-scan (*SADABS*; Sheldrick, 2004)	*h* = −22→22
*T*_min_ = 0.981, *T*_max_ = 0.983	*k* = −9→9
14877 measured reflections	*l* = −22→21

### Refinement

**Table:**

Refinement on *F*^2^	Primary atom site location: structure-invariant direct methods
Least-squares matrix: full	Secondary atom site location: difference Fourier map
*R*[*F*^2^ > 2σ(*F*^2^)] = 0.070	Hydrogen site location: inferred from neighbouring sites
*wR*(*F*^2^) = 0.147	H atoms treated by a mixture of independent and constrained refinement
*S* = 1.02	*w* = 1/[σ^2^(*F*_o_^2^) + (0.0535*P*)^2^ + 0.2161*P*] where *P* = (*F*_o_^2^ + 2*F*_c_^2^)/3
4994 reflections	(Δ/σ)_max_ < 0.001
329 parameters	Δρ_max_ = 0.19 e Å^−^^3^
8 restraints	Δρ_min_ = −0.18 e Å^−^^3^

### Special details

**Table:**

Geometry. All e.s.d.'s (except the e.s.d. in the dihedral angle between two l.s. planes) are estimated using the full covariance matrix. The cell e.s.d.'s are taken into account individually in the estimation of e.s.d.'s in distances, angles and torsion angles; correlations between e.s.d.'s in cell parameters are only used when they are defined by crystal symmetry. An approximate (isotropic) treatment of cell e.s.d.'s is used for estimating e.s.d.'s involving l.s. planes.
Refinement. Refinement of *F*^2^ against ALL reflections. The weighted *R*-factor *wR* and goodness of fit *S* are based on *F*^2^, conventional *R*-factors *R* are based on *F*, with *F* set to zero for negative *F*^2^. The threshold expression of *F*^2^ > σ(*F*^2^) is used only for calculating *R*-factors(gt) *etc*. and is not relevant to the choice of reflections for refinement. *R*-factors based on *F*^2^ are statistically about twice as large as those based on *F*, and *R*- factors based on ALL data will be even larger.

### Fractional atomic coordinates and isotropic or equivalent isotropic displacement parameters (Å^2^)

**Table:**

	*x*	*y*	*z*	*U*_iso_*/*U*_eq_	
N1	0.04236 (11)	0.8041 (3)	0.03832 (12)	0.0382 (5)	
N2	0.11383 (11)	0.8825 (3)	0.04497 (12)	0.0384 (6)	
N3	0.23967 (15)	1.1713 (4)	0.24440 (16)	0.0695 (8)	
N4	0.52913 (12)	0.2797 (3)	1.02064 (12)	0.0413 (6)	
N5	0.52601 (12)	0.3436 (3)	1.09279 (13)	0.0448 (6)	
N6	0.7097 (2)	0.4674 (6)	1.28277 (19)	0.1359 (17)	
O1	−0.07228 (11)	0.6992 (3)	0.11129 (11)	0.0655 (6)	
H1	−0.0294	0.7422	0.1060	0.098*	
O2	0.09556 (10)	0.9093 (3)	0.16978 (10)	0.0584 (6)	
O3	0.61680 (9)	0.1973 (3)	0.91423 (10)	0.0626 (6)	
H3A	0.6061	0.2342	0.9560	0.094*	
O4	0.65614 (10)	0.3548 (3)	1.10066 (11)	0.0614 (6)	
O5	0.10243 (14)	0.9509 (4)	0.32426 (12)	0.0823 (8)	
O6	0.76091 (12)	0.0749 (3)	0.05910 (11)	0.0669 (7)	
C1	−0.06363 (13)	0.6818 (3)	−0.02411 (15)	0.0364 (6)	
C2	−0.10201 (15)	0.6524 (4)	0.04338 (17)	0.0452 (7)	
C3	−0.17375 (16)	0.5708 (4)	0.04299 (19)	0.0577 (8)	
H3	−0.1992	0.5539	0.0878	0.069*	
C4	−0.20720 (16)	0.5150 (4)	−0.0231 (2)	0.0608 (9)	
H4	−0.2546	0.4582	−0.0225	0.073*	
C5	−0.17115 (17)	0.5424 (4)	−0.0898 (2)	0.0580 (9)	
H5C	−0.1940	0.5052	−0.1346	0.070*	
C6	−0.10039 (15)	0.6262 (4)	−0.08989 (16)	0.0480 (8)	
H6	−0.0766	0.6460	−0.1354	0.058*	
C7	0.01282 (13)	0.7672 (3)	−0.02630 (15)	0.0345 (6)	
C8	0.05050 (15)	0.8057 (4)	−0.09932 (15)	0.0515 (8)	
H8A	0.0888	0.8957	−0.0921	0.077*	
H8B	0.0125	0.8457	−0.1350	0.077*	
H8C	0.0744	0.7004	−0.1177	0.077*	
C9	0.13657 (14)	0.9254 (4)	0.11518 (17)	0.0406 (7)	
C10	0.21757 (14)	0.9977 (4)	0.12086 (16)	0.0496 (8)	
H10A	0.2539	0.9009	0.1186	0.060*	
H10B	0.2267	1.0749	0.0786	0.060*	
C11	0.23035 (14)	1.0954 (4)	0.19022 (18)	0.0471 (8)	
C12	0.47824 (13)	0.1629 (3)	0.90922 (15)	0.0389 (7)	
C13	0.55132 (15)	0.1436 (4)	0.87800 (15)	0.0465 (8)	
C14	0.55919 (17)	0.0688 (4)	0.80791 (17)	0.0619 (9)	
H14	0.6080	0.0552	0.7882	0.074*	
C15	0.4960 (2)	0.0143 (4)	0.76702 (17)	0.0643 (9)	
H15	0.5023	−0.0372	0.7202	0.077*	
C16	0.42393 (19)	0.0356 (4)	0.79496 (19)	0.0624 (9)	
H16	0.3810	0.0012	0.7669	0.075*	
C17	0.41539 (16)	0.1081 (4)	0.86462 (18)	0.0536 (8)	
H17	0.3660	0.1215	0.8831	0.064*	
C18	0.46728 (14)	0.2340 (3)	0.98523 (15)	0.0400 (7)	
C19	0.38820 (15)	0.2469 (5)	1.01681 (17)	0.0688 (10)	
H19A	0.3919	0.2576	1.0703	0.103*	
H19B	0.3595	0.1427	1.0040	0.103*	
H19C	0.3625	0.3487	0.9964	0.103*	
C20	0.59330 (16)	0.3755 (4)	1.12859 (15)	0.0443 (7)	
C21	0.58370 (16)	0.4428 (4)	1.20823 (15)	0.0546 (8)	
H21A	0.5595	0.5582	1.2065	0.066*	
H21B	0.5499	0.3637	1.2348	0.066*	
C22	0.6557 (2)	0.4559 (5)	1.24854 (19)	0.0759 (11)	
H2	0.1463 (13)	0.880 (4)	0.0059 (11)	0.080*	
H5	0.4823 (10)	0.368 (4)	1.1171 (15)	0.080*	
H5A	0.1041 (17)	0.937 (4)	0.2772 (6)	0.080*	
H6A	0.7337 (16)	0.163 (3)	0.0707 (15)	0.080*	
H5B	0.1286 (16)	1.039 (3)	0.3379 (15)	0.080*	
H6B	0.7723 (17)	0.019 (3)	0.0987 (11)	0.080*	

### Atomic displacement parameters (Å^2^)

**Table:**

	*U*^11^	*U*^22^	*U*^33^	*U*^12^	*U*^13^	*U*^23^
N1	0.0374 (12)	0.0357 (14)	0.0415 (15)	0.0014 (10)	−0.0027 (10)	0.0008 (12)
N2	0.0390 (12)	0.0451 (15)	0.0310 (15)	0.0014 (10)	−0.0022 (10)	−0.0015 (12)
N3	0.0771 (19)	0.074 (2)	0.057 (2)	−0.0111 (15)	−0.0134 (15)	−0.0057 (17)
N4	0.0430 (13)	0.0508 (15)	0.0303 (14)	0.0010 (11)	0.0024 (10)	0.0005 (12)
N5	0.0449 (14)	0.0549 (16)	0.0348 (15)	0.0034 (12)	0.0067 (11)	−0.0013 (13)
N6	0.092 (3)	0.233 (5)	0.082 (3)	−0.017 (3)	−0.021 (2)	−0.051 (3)
O1	0.0614 (13)	0.0916 (18)	0.0436 (14)	−0.0126 (12)	0.0070 (10)	0.0058 (12)
O2	0.0450 (11)	0.0912 (17)	0.0389 (13)	−0.0068 (10)	0.0018 (9)	−0.0022 (11)
O3	0.0386 (10)	0.1050 (18)	0.0441 (13)	0.0030 (11)	0.0005 (9)	−0.0131 (13)
O4	0.0458 (11)	0.0855 (17)	0.0530 (14)	−0.0069 (11)	0.0077 (9)	−0.0082 (12)
O5	0.0884 (17)	0.108 (2)	0.0508 (15)	−0.0382 (14)	0.0206 (13)	−0.0045 (15)
O6	0.0567 (13)	0.090 (2)	0.0542 (15)	0.0036 (11)	0.0051 (11)	0.0093 (13)
C1	0.0382 (14)	0.0330 (16)	0.0378 (17)	0.0080 (12)	−0.0024 (12)	0.0038 (14)
C2	0.0454 (15)	0.0450 (19)	0.045 (2)	0.0040 (14)	−0.0027 (14)	0.0052 (16)
C3	0.0477 (17)	0.060 (2)	0.065 (2)	−0.0003 (15)	0.0080 (16)	0.0150 (19)
C4	0.0408 (16)	0.049 (2)	0.092 (3)	−0.0020 (14)	−0.0071 (18)	0.009 (2)
C5	0.0531 (18)	0.053 (2)	0.067 (2)	−0.0006 (15)	−0.0208 (17)	−0.0009 (18)
C6	0.0485 (16)	0.0470 (19)	0.048 (2)	0.0045 (14)	−0.0059 (14)	0.0026 (15)
C7	0.0385 (14)	0.0317 (16)	0.0334 (17)	0.0075 (11)	−0.0017 (12)	0.0033 (13)
C8	0.0453 (15)	0.067 (2)	0.0417 (19)	−0.0043 (14)	−0.0018 (13)	0.0024 (16)
C9	0.0366 (14)	0.0436 (18)	0.0415 (19)	0.0054 (12)	−0.0011 (13)	0.0041 (15)
C10	0.0384 (15)	0.066 (2)	0.045 (2)	0.0052 (14)	−0.0032 (13)	−0.0046 (16)
C11	0.0392 (15)	0.055 (2)	0.047 (2)	−0.0027 (13)	−0.0065 (14)	0.0077 (17)
C12	0.0426 (15)	0.0383 (17)	0.0357 (17)	−0.0015 (12)	−0.0007 (12)	0.0086 (14)
C13	0.0465 (16)	0.060 (2)	0.0327 (18)	0.0011 (14)	−0.0028 (13)	0.0050 (16)
C14	0.0600 (19)	0.087 (3)	0.038 (2)	0.0016 (17)	0.0045 (15)	−0.0049 (19)
C15	0.090 (3)	0.070 (2)	0.033 (2)	−0.0125 (19)	−0.0023 (18)	0.0001 (17)
C16	0.071 (2)	0.064 (2)	0.052 (2)	−0.0215 (18)	−0.0126 (17)	0.0075 (19)
C17	0.0477 (17)	0.061 (2)	0.052 (2)	−0.0074 (15)	−0.0003 (15)	0.0073 (18)
C18	0.0392 (15)	0.0393 (18)	0.0414 (18)	0.0016 (12)	0.0012 (13)	0.0083 (14)
C19	0.0436 (17)	0.098 (3)	0.065 (2)	−0.0001 (17)	0.0098 (15)	−0.010 (2)
C20	0.0498 (17)	0.0450 (19)	0.0383 (18)	−0.0031 (14)	0.0052 (14)	0.0070 (15)
C21	0.0664 (19)	0.057 (2)	0.0404 (19)	−0.0009 (16)	0.0021 (15)	−0.0048 (16)
C22	0.074 (2)	0.105 (3)	0.049 (2)	−0.012 (2)	0.0000 (19)	−0.021 (2)

### Geometric parameters (Å, °)

**Table:**

N1—C7	1.286 (3)	C5—H5C	0.9300
N1—N2	1.381 (3)	C6—H6	0.9300
N2—C9	1.348 (3)	C7—C8	1.498 (3)
N2—H2	0.904 (10)	C8—H8A	0.9600
N3—C11	1.135 (3)	C8—H8B	0.9600
N4—C18	1.286 (3)	C8—H8C	0.9600
N4—N5	1.378 (3)	C9—C10	1.513 (3)
N5—C20	1.346 (3)	C10—C11	1.457 (4)
N5—H5	0.901 (10)	C10—H10A	0.9700
N6—C22	1.115 (4)	C10—H10B	0.9700
O1—C2	1.357 (3)	C12—C13	1.404 (3)
O1—H1	0.8200	C12—C17	1.404 (4)
O2—C9	1.224 (3)	C12—C18	1.476 (3)
O3—C13	1.362 (3)	C13—C14	1.383 (4)
O3—H3A	0.8200	C14—C15	1.372 (4)
O4—C20	1.219 (3)	C14—H14	0.9300
O5—H5A	0.848 (10)	C15—C16	1.367 (4)
O5—H5B	0.845 (10)	C15—H15	0.9300
O6—H6A	0.848 (10)	C16—C17	1.370 (4)
O6—H6B	0.843 (10)	C16—H16	0.9300
C1—C6	1.393 (3)	C17—H17	0.9300
C1—C2	1.405 (4)	C18—C19	1.498 (3)
C1—C7	1.480 (3)	C19—H19A	0.9600
C2—C3	1.392 (4)	C19—H19B	0.9600
C3—C4	1.373 (4)	C19—H19C	0.9600
C3—H3	0.9300	C20—C21	1.523 (4)
C4—C5	1.372 (4)	C21—C22	1.437 (4)
C4—H4	0.9300	C21—H21A	0.9700
C5—C6	1.384 (4)	C21—H21B	0.9700
			
C7—N1—N2	121.1 (2)	C11—C10—H10A	109.3
C9—N2—N1	115.7 (2)	C9—C10—H10A	109.3
C9—N2—H2	123.0 (19)	C11—C10—H10B	109.3
N1—N2—H2	119.9 (19)	C9—C10—H10B	109.3
C18—N4—N5	120.6 (2)	H10A—C10—H10B	107.9
C20—N5—N4	117.4 (2)	N3—C11—C10	179.4 (3)
C20—N5—H5	117.9 (19)	C13—C12—C17	116.4 (3)
N4—N5—H5	124.7 (19)	C13—C12—C18	122.3 (2)
C2—O1—H1	109.5	C17—C12—C18	121.2 (2)
C13—O3—H3A	109.5	O3—C13—C14	117.2 (2)
H5A—O5—H5B	111 (2)	O3—C13—C12	122.4 (3)
H6A—O6—H6B	108 (2)	C14—C13—C12	120.4 (3)
C6—C1—C2	117.2 (2)	C15—C14—C13	121.0 (3)
C6—C1—C7	120.6 (2)	C15—C14—H14	119.5
C2—C1—C7	122.1 (2)	C13—C14—H14	119.5
O1—C2—C3	116.6 (3)	C16—C15—C14	120.1 (3)
O1—C2—C1	123.1 (2)	C16—C15—H15	120.0
C3—C2—C1	120.3 (3)	C14—C15—H15	120.0
C4—C3—C2	120.5 (3)	C15—C16—C17	119.5 (3)
C4—C3—H3	119.7	C15—C16—H16	120.2
C2—C3—H3	119.7	C17—C16—H16	120.2
C5—C4—C3	120.5 (3)	C16—C17—C12	122.6 (3)
C5—C4—H4	119.8	C16—C17—H17	118.7
C3—C4—H4	119.8	C12—C17—H17	118.7
C4—C5—C6	119.3 (3)	N4—C18—C12	115.5 (2)
C4—C5—H5C	120.4	N4—C18—C19	124.2 (3)
C6—C5—H5C	120.4	C12—C18—C19	120.2 (2)
C5—C6—C1	122.2 (3)	C18—C19—H19A	109.5
C5—C6—H6	118.9	C18—C19—H19B	109.5
C1—C6—H6	118.9	H19A—C19—H19B	109.5
N1—C7—C1	114.6 (2)	C18—C19—H19C	109.5
N1—C7—C8	124.4 (2)	H19A—C19—H19C	109.5
C1—C7—C8	121.0 (2)	H19B—C19—H19C	109.5
C7—C8—H8A	109.5	O4—C20—N5	124.1 (3)
C7—C8—H8B	109.5	O4—C20—C21	122.6 (3)
H8A—C8—H8B	109.5	N5—C20—C21	113.3 (2)
C7—C8—H8C	109.5	C22—C21—C20	112.5 (3)
H8A—C8—H8C	109.5	C22—C21—H21A	109.1
H8B—C8—H8C	109.5	C20—C21—H21A	109.1
O2—C9—N2	123.5 (2)	C22—C21—H21B	109.1
O2—C9—C10	122.5 (3)	C20—C21—H21B	109.1
N2—C9—C10	114.1 (2)	H21A—C21—H21B	107.8
C11—C10—C9	111.7 (2)	N6—C22—C21	176.7 (4)

### Hydrogen-bond geometry (Å, °)

**Table:**

*D*—H···*A*	*D*—H	H···*A*	*D*···*A*	*D*—H···*A*
O6—H6B···N6^i^	0.84 (1)	2.17 (2)	2.975 (4)	159 (3)
O5—H5B···O3^ii^	0.85 (1)	2.43 (2)	3.120 (4)	140 (3)
O6—H6A···O4^iii^	0.85 (1)	2.06 (1)	2.900 (3)	173 (3)
O5—H5A···O2	0.85 (1)	1.93 (1)	2.777 (3)	174 (3)
N5—H5···O5^iv^	0.90 (1)	1.93 (1)	2.820 (3)	171 (3)
N2—H2···O6^v^	0.90 (1)	2.03 (1)	2.905 (3)	162 (3)
O3—H3A···N4	0.82	1.82	2.534 (3)	145.
O1—H1···N1	0.82	1.81	2.528 (3)	145.

Symmetry codes: (i) −*x*+3/2, *y*−1/2, −*z*+3/2; (ii) *x*−1/2, −*y*+3/2, *z*−1/2; (iii) *x*, *y*, *z*−1; (iv) −*x*+1/2, *y*−1/2, −*z*+3/2; (v) −*x*+1, −*y*+1, −*z*.
